# Antibacterial Activity of Hexanol Vapor In Vitro and on the Surface of Vegetables

**DOI:** 10.3390/foods12163097

**Published:** 2023-08-17

**Authors:** Daisuke Kyoui, Yuka Saito, Akifumi Takahashi, Gou Tanaka, Runa Yoshida, Yoshiyuki Maegaki, Taketo Kawarai, Hirokazu Ogihara, Chise Suzuki

**Affiliations:** Laboratory of Food Microbiology, Department of Food Science and Technology, College of Bioresource Sciences, Nihon University, 1866 Kameino, Fujisawa 2520880, Kanagawa, Japan

**Keywords:** antibacterial treatment, hexanol, vapor, vegetable freshness

## Abstract

Hexanol is a volatile alcohol and a major component of plant essential oils (EOs). However, the antibacterial activity of hexanol vapor has not been well studied. This study aimed to evaluate the antibacterial activity of hexanol. In this study, seven food-related bacteria were exposed to 1-, 2- or 3-hexanol vapor on agar media to evaluate their growth. Additionally, the total viable counts in three vegetables when exposed to 1-hexanol vapor were measured. The results showed that 1-hexanol exhibited antibacterial effects against Gram-negative bacteria but did not affect Gram-positive bacteria. However, compounds 2- and 3-hexanol did not show antimicrobial activity against any bacteria. For the vegetables, exposure to 1-hexanol vapor decreased the total viable bacterial counts in cabbage and carrot and inhibited bacterial growth in eggplants. In cabbage, 1-hexanol vapor at concentrations over 50 ppm decreased the total viable count within 72 h, and 25 ppm of vapor showed bacteriostatic activity for 168 h. However, 1-hexanol vapor also caused discoloration in cabbage. In summary, 1-hexanol has the potential to act as an antibacterial agent, but further studies are required for practical use. Moreover, the study results may help determine the antimicrobial activity of various EOs in the future.

## 1. Introduction

Essential oils (EOs) are volatile plant extracts generally regarded as ecologically friendly and consumer-acceptable antimicrobial agents used for food preservation [1]. EOs are mixtures of various compounds such as terpenes, phenols, aldehydes, alcohols, and ketones [2]; antimicrobial compounds have also been identified in EOs. For example, carvacrol and thymol, which are contained in herbs such as coriander, oregano, and thyme EOs, are representative of antibacterial compounds in EOs [3]. Hexanol (CH_3_(CH)_5_OH) is a volatile alcohol with a green leaf flavor and is a major component of EOs extracted from cherry, guava, olive, and perilla [4,5,6,7,8]. EOs extracted from buckwheat flowers (*Fagopyrum esculentum*/*tataricum*/*cymosum)*, which contain 2.40–7.07% 1-hexanol, showed antimicrobial activity against *Agrobacterium tumefaciens*, *Escherichia coli*, *Pseudomonas lachrymans Xanthomonas vesicatoria*, *Bacillus subtilis*, and *Staphylococcus aureus* with a minimum inhibitory concentration (MIC) value of 200–800 µg/mL [9]. Moreover, coastal coral trees (*Erythrina caffra*) EOs, which include 3.31% 1-hexanol, showed antimicrobial activity against *B. cereus*, *B. subtilis*, *Enterococcus faecalis*, *E. coli*, *Shigella sonnei*, *Shi. flexneri*, *Staph. aureus*, *Streptococcus pyogenes*, *Proteus vulgaris*, and *Serratia marcescens* following an agar diffusion test [10]. These findings suggest that hexanol may play a role in the antimicrobial activity of EOs.

The antimicrobial activity of hexanol has previously been reported. *E. coli* delayed its growth when over 3.9 mM of hexanol was added to the liquid medium directly [11,12]. Additionally, its antimicrobial activity against *B. subtilis*, *Propionibacterium acnes*, *Pitylosporum ovale*, and *Acinetobacter calcoaceticusi* has been reported [12,13,14]. On the other hand, other groups have reported that hexanol did not show antimicrobial activity against bacteria, including *E. coli* and *B. subtilis,* when hexanol, dissolved in dimethylformamide, was added to the liquid medium adjusted to 800 µg/mL [13,15,16]. Although these studies provide some information about the antimicrobial activity of hexanol, comprehensive knowledge about the antimicrobial activity of hexanol has not yet been determined because the conditions, such as the dilution of hexanol and evaluation of antimicrobial activity, differ between studies. In particular, hexanol remains almost completely undissolved in water owing to its hydrophobicity, and thus diffusion and dissolution methods may directly affect the results.

EOs are often added to foods directly but other methods are also used to protect EOs from inactivation caused by interaction with food components and to improve the contact of EOs to the target microbial. For example, emulsions and encapsulations can improve the stability and functionality of EOs [17,18]. Another example includes edible films and coatings containing EOs, which can protect food from the invasion of microorganisms via the atmosphere [17]. Vapor phase EOs is also an additive method, which has some advantages, such as a small effect on the organoleptic properties of food and high diffusibility [19]. Furthermore, some studies have reported that EO vapor shows higher antimicrobial activity than liquid-phase EO [17,20]. In this study, the antimicrobial activity of the vapor phase of hexanol was investigated to recognize the antimicrobial activity of EO vapor. We evaluated the antimicrobial activity of hexanol isomers against food-related bacteria. Moreover, the antimicrobial activity of 1-hexanol against the total viable counts of vegetables was evaluated. The results could help determine the antimicrobial activity of hexanol and be useful in realizing the antimicrobial activity of EOs as well as in using EOs for food treatment.

## 2. Materials and Methods

### 2.1. Exposure Test of Microorganisms on an Agar Medium

*Escherichia coli* IAM1264 (Ec), *Pseudomonas aeruginosa* ATCC 10145 (Pa), *Salmonella enterica* Enteritidis IFO 3313 (SE), *Bacillus subtilis* ATCC11774 (Bs), *Lactiplantibacillus plantarum* ATCC8014 (Lp), *Listeria monocytogenes* ATCC15313 (Lm), and *Staphylococcus aureus* ATCC 12600 (Sa) were used for the exposure test. Ec, Pa, SE, Bs, Lm, and Sa were inoculated in Trypticase Soy Broth (Becton Dickinson and Company, Franklin Lakes, NJ, USA). Lp was inoculated into Lactobacilli MRS Broth (Becton Dickinson and Company). The culture temperature was set to 37 °C for Ec, SE, Lm, and Sa; 30 °C for Lp; and 20 °C for Pa. The cells were cultured overnight, and then one loop of the culture medium was inoculated into a fresh medium and cultured under the same conditions.

For the exposure test, Trypticase Soy Agar (TSA; Becton Dickinson and Company) was used as the medium for Ec, Pa, SE, Bs, Lm, and Sa, and MRS agar (Becton Dickinson and Company) for Lp. The 20 mL of medium was poured into a Petri dish (90 φ × 15 mm; polystyrene), resulting in a head space of 75.35 mL. 

Three hexanol isomers (1-, 2-, and 3-hexanol; FUJIFILM Wako Pure Chemical Corporation, Osaka, Japan) were used in this study. Previous studies did not define the isomers of hexanol for examination [11,12]. It was expected that a comparison of the antimicrobial activity of these isomers would provide information regarding their antimicrobial mechanisms. The hexanols were diluted in ethanol to 452, 301, 150, 75, and 0 μL/L to achieve headspace concentrations of 300, 200, 100, 50, and 0 ppm, respectively, when 50 µL of diluted hexanol was injected.

A total of 100 µL of pre-cultured medium was spread onto the medium for the exposure test, and the Petri dish was inverted. Thereafter, a filter paper (φ 8 mm; Advantec Toyo Kaisha, Ltd., Tokyo, Japan) was fixed to the center inside the Petri dish cover. The filter paper was then soaked in each diluted hexanol (50 µL). The Petri dish was closed immediately and sealed using laboratory film. The Petri dishes were cultured at 37 °C for Ec, SE, Bs, Lm, and Sa; 20 °C for Pa; and 30 °C for Bs and Lp, for 48 h. Following incubation, the sensitivity of the cultured microorganisms to hexanol was evaluated. The effect was categorized into three types: “the distinct clear zone was defined (++)”, “the clear zone was not defined but growth was delayed and colony density was low (+)”, and “no differences were observed compared to the control (−)”. The evaluation was repeated twice.

### 2.2. Exposure Test for Vegetables

The test vegetables used in this study were cabbage, carrot, and eggplant, collected from supermarkets in Tokyo and Kanagawa prefectures between April 2021 to October 2022. The carrots and eggplants were peeled, and the peels and cabbage leaves were cut into approximately 1 cm square pieces.

Three stainless steel trays were placed in a 2.5 L polycarbonate case (Sugiyamagen Co., Ltd., Tokyo, Japan). Ten grams of the sample was placed in one of the trays. In the second tray, absorbent cotton was placed, and 750 µL of 1-hexanol was soaked to concentrate the atmosphere in the polycarbonate case to 300 ppm. Distilled water was used in place of 1-hexanol as the control. In the third tray, 20 mL of a saturated phosphate dihydro ammonium solution was added to maintain a humidity of approximately 93%. The polycarbonate case was then sealed and incubated at 20 °C for 24 h. 

For the cabbage, its total viable counts were decreased significantly. Additionally, cabbage leaf included the edible part, while carrot peel was not. Therefore, extra experiments were conducted for cabbage in the present study. For the cabbage, the concentration of 1-hexanol was modified to 100, 50, and 25 ppm, and the incubation time was set for 0, 24, 72, and 168 h using the same conditions. These examinations were repeated three times.

Following incubation, the samples were recovered and 90 mL of buffered saline (0.85% NaCl, aq.) was added. The mixture was homogenized and the homogenates were then serially diluted and spread on TSA. The medium was incubated at 30 °C for 48 h, and the colonies were counted. 

### 2.3. Colorimetric Measurement of Cabbage Exposed to 1-Hexanol

Cabbage was exposed to 100 or 25 ppm of 1-hexanol for 168 h, as described in Section 2.2. Distilled water was used in place of 1-hexanol as the control. After storage, the cabbage color was measured using a color-difference meter (CR-200, KONICA MINOLTA, Inc., Tokyo, Japan). Colorimetric measurements were performed using the Lab color space, and five points in each sample were randomly selected for the measurements. This examination was repeated twice.

### 2.4. Statistical Analysis

The results of the exposure test for vegetables and colorimetric changes were compared between the conditions using a one-way ANOVA and the Bonferroni–Dann test.

## 3. Results

### 3.1. Sensitivity of Food-Related Bacteria to Hexanol Isomer Vapor

Exposure to 1-hexanol vapor inhibited the growth of Ec, Pa, and SE at concentrations greater than 150 ppm (Table 1). In contrast, Bs, Lp, Lm, and Sa were not sensitive to 1-hexanol. The vapors of 2- and 3-hexanol did not exhibit antimicrobial activity against food-related bacteria.

### 3.2. Effect of 1-Hexanol Vapor on the Viable Counts in Vegetables

**Figure 1:**
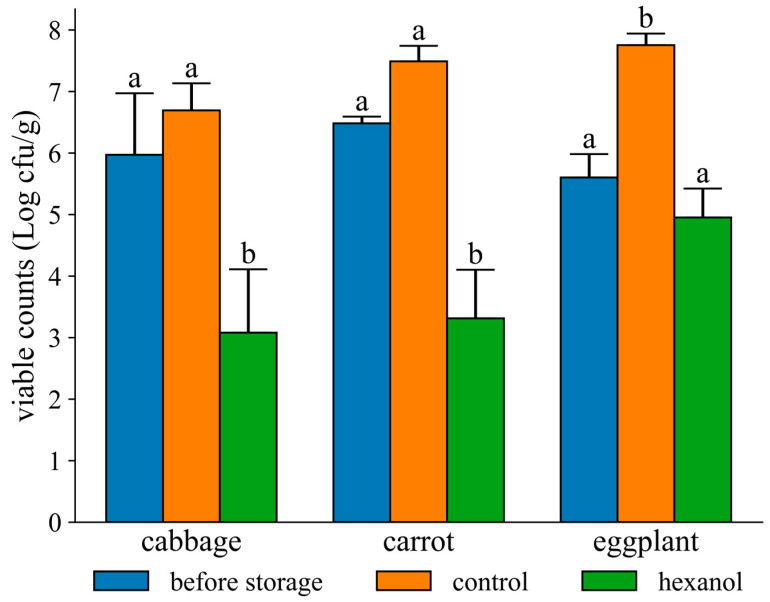
Viable counts in vegetables following exposure to 300 ppm of 1-hexanol vapor. The letters on the bars indicate the significance tested using the Bonferroni–Dunn test.

The viable counts in cabbage and carrot decreased by approximately 3 Logs after 24 h of storage with exposure to 300 ppm of 1-hexanol vapor (Figure 1). These viable counts were significantly lower than those before storage or under control storage conditions (*p* < 0.05). In eggplants, the viable counts did not change significantly after storage with 300 ppm of 1-hexanol vapor. However, under control conditions, the viable counts increased significantly compared to those before storage.

### 3.3. Changes in the Viable Counts of Cabbage under Storage with 1-Hexanol Vapor

**Figure 2:**
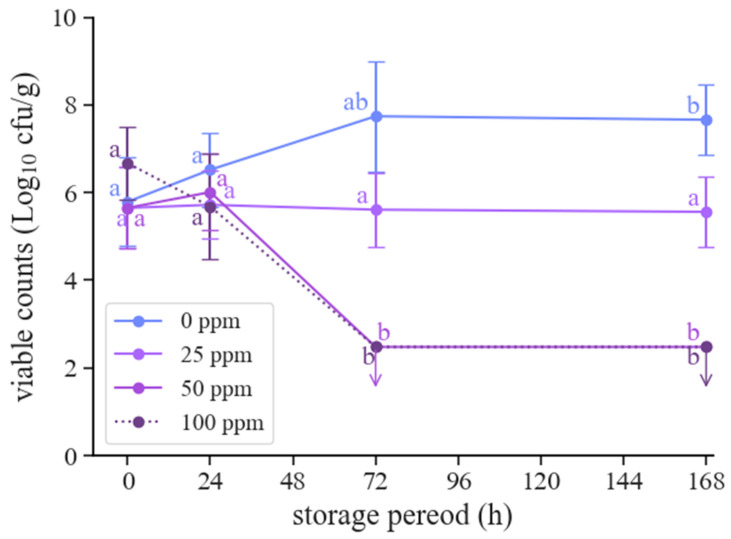
Viable counts of cabbage during storage after exposure to various concentrations of 1-hexanol vapor. The arrows indicate that the viable counts were below the detection limit. The letters show significance tested using the Bonferroni–Dunn test between the storage periods for each condition.

Changes in the viable counts of cabbage exposed to 0–100 ppm of 1-hexanol vapor were examined (Figure 2). When stored without 1-hexanol, the viable cell counts increased until 168 h. In contrast, upon exposure to 25 ppm of 1-hexanol, the viable counts were stable after 72 h of storage. In addition, exposure to >50 ppm of 1-hexanol decreased the viable counts to levels below the detection limit after 72 h of storage.

### 3.4. Effect of 1-Hexanol on the Colorimetric Measurements of Cabbage

Colorimetric measurements were performed on the cabbage before and after 168 h of storage with exposure to 25 or 100 ppm of 1-hexanol vapor (Table 2). The L-values remained unchanged after storage under both conditions. However, the a-value increased significantly after storage with 1-hexanol vapor, reaching −5.42 ± 1.50 and −4.90 ± 1.81 when stored at 25 and 100 ppm concentrations, respectively, compared to −10.95 ± 2.12 before storage and −12.17 ± 1.80 after storage without exposure to 1-hexanol vapor. The b-value decreased slightly, although not significantly, from 21.77 ± 1.11 before storage to 19.14 ± 1.70 and 18.25 ± 1.79 when stored with 25 and 100 ppm of 1-hexanol vapor, respectively. These changes in the a- and b-values indicate that the green-yellow color of the cabbage turned reddish and discolored, respectively. 

## 4. Discussion

This study examined the antimicrobial activity of 1-hexanol against food-related bacteria. The sensitivity to 1-hexanol varied depending on the Gram-staining ability of the bacteria (Table 1). Similarly, essential oil extracted from *Phrynium pubinerve* containing 8.61% 1-hexanol showed lower MICs of *E. coli* and *Klebsiella pneumoniae* than *S. aureus* [21]. It has been suggested that alcohols increase the fluidity of the cell membrane, leading to the deconstruction of the membrane and proteins [22,23]. However, our results did not support this hypothesis, as Gram-positive bacteria were found to be insensitive to 1-hexanol. Instead, we postulated that 1-hexanol affects the outer membrane of Gram-negative bacteria. Lipopolysaccharides, which make up half of the outer membrane, are usually hydrophobic owing to the presence of four to eight acyl groups and are almost saturated compared to phospholipids, which include two acyl groups [24]. We hypothesized that 1-hexanol, which is more hydrophobic than ethanol, enters the hydrophobic part of the lipopolysaccharide in the outer membrane, leading to its deconstruction. This hypothesis is supported by our finding that 2- and 3-hexanol, which have shorter hydrophobic chains than 1-hexanol, did not exhibit antimicrobial activity against Gram-negative bacteria. Long-chain alcohols, in contrast, have been reported to decrease the antimicrobial activity against Gram-negative bacteria, while exhibiting antimicrobial activity against Gram-positive bacteria [12]. Further research is required to clarify this relationship.

In this study, we examined the antimicrobial activity of 1-hexanol against food-related bacteria. The results showed that the growth of Gram-negative bacteria was inhibited by exposure to >150 ppm of 1-hexanol vapor (Table 1). This concentration was remarkably lower than that reported in previous studies, which showed that 800 µg/mL of 1-hexanol exhibited antimicrobial activity [12,13,25,26]. However, the addition method used in this study was 1-hexanol vapor, which differed from those used in previous studies in which hexanol was added to a liquid medium directly or dissolved in dimethylformamide. Furthermore, some EO vapors have been reported to be more effective than their liquid form [17], and highly hydrophobic EOs and hexanol may form micelles in water, thus preventing contact between the compounds and microorganisms [27]. 

In this study, cabbage, carrot, and eggplant, which represent leaf, root, and fruit vegetables, respectively, were used for the exposure examination (Figure 1). The results showed that 1-hexanol vapor decreased the viable counts in cabbage and carrots. Previous studies have reported that their microflora is mainly composed of Gram-negative bacteria [28,29]. As described earlier, Gram-negative bacteria that are sensitive to 1-hexanol were considered sterilized. On the other hand, the total viable counts in the eggplant were not changed when exposed to 1-hexanol but were increased under control conditions. The microflora of eggplant has not been explored, but Gram-positive and -negative bacteria would inhabit on its surface. Therefore, it was assumed that 1-hexanol-sensitive bacteria, which is mainly Gram-negative bacteria, decreased, whereas 1-hexanol-insensitive bacteria, which is mainly Gram-positive bacteria, increased. In summary, the apparent total viable counts were unchanged when stored with 1-hexanol vapor.

The time series examination of cabbage storage showed that the viable counts decreased after 72 h of storage with >50 ppm of 1-hexanol vapor (Figure 2). However, the count decreased within 24 h of storage with 300 ppm of 1-hexanol vapor (Figure 1). During the experiments, the sample was sealed and maintained at a static temperature. The diffusion of 1-hexanol vapor from the paper disc into the case would be faster at higher concentrations, according to Fick’s laws of diffusion. Therefore, the differences in storage time until the number of viable cells decreased were assumed to be dependent on the diffusion speed and concentration. Furthermore, 25 ppm of 1-hexanol vapor inhibited the growth of viable cells until 168 h of storage. In contrast, in the absence of 1-hexanol vapor, the viable cells increased 7.7 ± 0.8 Log cfu/g for 168 h. Conventionally, sensory indicators such as odor, taste, or visual cues suggest that food is deemed spoiled when viable counts reach approximately 7 Log organisms/g [30]. Thus, it can be inferred that 1-hexanol has potential as an agent in the preservation treatment of vegetables. However, certain challenges need to be addressed, as detailed below.

Exposure of cabbage to 1-hexanol vapor caused green discoloration (Table 2). This is because the green color of plants is mainly derived from chlorophyll, and chlorophyll a, b, d, and f are lipophilic and easily drained off by organic solvents [31]. In this study, dripping was observed in the cabbage stored with 1-hexanol vapors (the dripping was not quantified. Therefore, the 1-hexanol vapor caused leakage from the cabbage, leading to the removal of chlorophyll and subsequent discoloration. This can be considered a disadvantage of the 1-hexanol vapor treatment of vegetables and should be addressed in future research.

## 5. Conclusions

The present study aimed to validate the antimicrobial activity of 1-hexanol vapor. In this study, it was revealed that the antimicrobial sensitivity is species-dependent and differs between Gram-positive and -negative bacteria. The observations in this study partly revealed the antimicrobial activity of EOs. On the other hand, 1-hexanol exhibits potential as a food preservative, in particular, because it has a green leaf-like smell, which makes it likely to be used for treatment because of its low sensory impact. However, further research is needed such as inoculation experiment of pathogenic bacteria on a vegetable. Additionally, it has not been listed on the positive list by major government organizations, such as FDA, EFSA, and MHLW, although some EOs are generally recognized as safe (GRAS). Further research about the detailed antimicrobial activity and safety is required to use purified 1-hexanol as a food additive.

## Figures and Tables

**Table 1:** Sensitivity of food-related bacteria to hexanols.

	Concentration (ppm)											
	1-Hexanol				2-Hexanol				3-Hexanol			
Strain	300	150	75	38	300	150	75	38	300	150	75	38
*Escherichia coli* IAM 1264	++	+	−	−	−	−	−	−	−	−	−	−
*Pseudomonas aeruginosa* ATCC 10145	++	+	−	−	−	−	−	−	−	−	−	−
*Salmonella enterica Enteritidis* IFO 3313	++	++	−	−	−	−	−	−	−	−	−	−
*Bacillus subtilis* ATCC 11774	−	−	−	−	−	−	−	−	−	−	−	−
*Lactiplantibacillus plantarum* ATCC 8014	−	−	−	−	−	−	−	−	−	−	−	−
*Listeria monocytogenes* ATCC 15313	−	−	−	−	−	−	−	−	−	−	−	−
*Staphylococcus aureus* ATCC 12600	−	−	−	−	−	−	−	−	−	−	−	−

++—a distinct clear zone was observed; +—the growth was inhibited −—any effect was not observed.

**Table 2:** Lab color space values of cabbage exposed to 1-hexanol.

	L	a	b
before storage	70.2 ± 7.0 a	−10.95 ± 2.12 a	21.77 ± 1.11 ab
0 ppm	76.4 ± 6.2 a	−12.17 ± 1.80 a	23.95 ± 1.03 a
25 ppm	73.1 ± 4.9 a	−5.42 ± 1.50 b	19.14 ± 1.70 ab
100 ppm	70.9 ± 5.6 a	−4.90 ± 1.81 b	18.25 ± 1.79 b

Letters indicate significance (*p* < 0.05) using the Bonferroni–Dann test.

## Data Availability

Data is contained within the article.
